# Trans-generational epigenetic regulation of *C. elegans *primordial germ cells

**DOI:** 10.1186/1756-8935-3-15

**Published:** 2010-08-12

**Authors:** Hirofumi Furuhashi, Teruaki Takasaki, Andreas Rechtsteiner, Tengguo Li, Hiroshi Kimura, Paula M Checchi, Susan Strome, William G Kelly

**Affiliations:** 1 Biology Department, Emory University, Atlanta, GA 30322, USA; 2 Department of MCD Biology, University of California, Santa Cruz, Santa Cruz, CA 95064, USA; 3 Graduate School for Frontier Biosciences, Osaka University, 1-3 Yamadaoka, Suita, Osaka 565-0871, Japan; 4 Graduate School of Pharmaceutical Sciences, Tohoku University, Sendai 980-8578, Japan; 5 Department of Molecular & Cellular Biology, UC Davis, Davis, CA 95616, USA

## Abstract

**Background:**

The processes through which the germline maintains its continuity across generations has long been the focus of biological research. Recent studies have suggested that germline continuity can involve epigenetic regulation, including regulation of histone modifications. However, it is not clear how histone modifications generated in one generation can influence the transcription program and development of germ cells of the next.

**Results:**

We show that the histone H3K36 methyltransferase maternal effect sterile (MES)-4 is an epigenetic modifier that prevents aberrant transcription activity in *Caenorhabditis elegans *primordial germ cells (PGCs). In *mes-4 *mutant PGCs, RNA Pol II activation is abnormally regulated and the PGCs degenerate. Genetic and genomewide analyses of MES-4-mediated H3K36 methylation suggest that MES-4 activity can operate independently of ongoing transcription, and may be predominantly responsible for maintenance methylation of H3K36 in germline-expressed loci.

**Conclusions:**

Our data suggest a model in which MES-4 helps to maintain an 'epigenetic memory' of transcription that occurred in germ cells of previous generations, and that MES-4 and its epigenetic product are essential for normal germ cell development.

## Background

Chromatin structure is an important determinant of transcriptional activity, and is thought to influence accessibility of the transcriptional machinery to the DNA and/or modulate its productivity, as a component of regulation. The structure of chromatin and its influence on genetic regulation can be heritable, and this heritability forms the basis of epigenetic forms of genome regulation. As the eukaryotic genome is passed between generations, there occurs significant remodeling or re-programming of the gamete epigenomes as they merge in the zygote. An additional round of epigenetic reprogramming also occurs upon establishment of the embryonic germline in many species [1]. The purpose of these events are not clear, but they are thought to be important for resetting an epigenetic 'ground state' that is compatible with developmental pluripotency in the zygote, and with maintaining or establishing totipotency in the germline. Although much of the research focus has been on epigenetic erasure events that occur in the zygote, it is important to note that significant epigenetic information is probably retained and/or re-established in the zygote and primordial germ cells (PGCs). How any epigenetic information is selected for erasure, retention or establishment is not yet understood.

Interestingly, a state of transcriptional quiescence also accompanies germline determination in many organisms [2]. In *Drosophila *and *C. elegans*, this quiescence is achieved by interfering with RNA polymerase (Pol) II transcriptional activation. A key mechanism of transcriptional regulation is phosphorylation of serine residues (specifically serine 2 and serine 5) within a highly repetitious seven amino acid sequence in the C-terminal domain (CTD) of the largest subunit of Pol II [3]. During the transition from initiation stages to productive elongation, Ser2 is phosphorylated by positive transcription elongation factor (P-TEFb), the predominant kinase complex that targets this residue in the CTD repeat [3,4]. In *C. elegans*, in addition to P-TEFb, Tousled-like kinase (TLK-1) has also been implicated in regulating phosphorylation of the CTD of Pol II [5].

**Figure 1:**
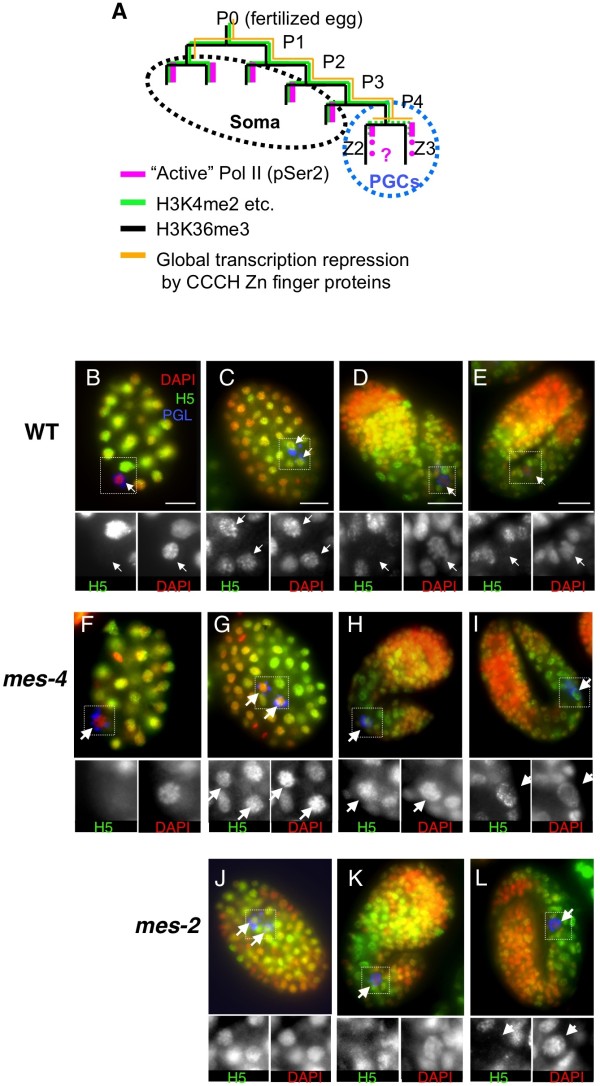
**Unique and dynamic regulation of Pol II in the embryonic germline**. **(A) **Transcription status and histone modification dynamics in *C. elegans *early embryonic blastomeres. The asymmetric divisions of P-lineage cells (P0 to P3) produce both germline and somatic blastomeres. The last P cell division (P4) is symmetric and produces two germline-committed primordial germ cells (PGCs), Z2 and Z3 and these cells arrest at G2 phase through the rest of embryogenesis [68]. Two maternally expressed CCCH zinc finger proteins, OMA-1/2 and PIE-1, act sequentially in P0 to P4 to maintain transcriptional quiescence independently of the chromatin environment [14,69,70] (orange line). At the P4 division, PIE-1 is degraded quickly and 'active Pol II' (H5 staining; magenta line) appears in the PGCs (circled by blue dotted line). H3K36me3 (black) and H3K4me2 (green) are initially present in both the transcriptionally quiescent P-lineage and their somatic sisters, where transcription is activated. MES-4, which is essential for germ cell viability, produces H3K36me in the germline precursors independently of transcription [21]. H3K4me2 is also maintained through the P-lineage by an unidentified mechanism, but the level of H3K4me2 becomes almost undetectable in the PGCs [15] (indicated by dotted green lines). **(B-L) **H5 staining of Pol II Ser2P in the PGCs of **(B-E) **wild type, **(F-I) ***mes-4 *and **(J-L) ***mes-2* during embryogenesis; **(B,F)**~150 minutes post-fertilization at 22°C, ~26-cell stage; **(C,G,J) **~200 minutes, ~90-cell stage; **(D,H,K) **~450 minutes, ~1.5-fold stage; **(E,I,L) **>500 minutes, 2- to 3-fold stages. PGCs are boxed and shown by arrows. DAPI staining, red; H5 antibody staining, green; PGL-1 (germline marker) staining, blue. In the lower panels, PGCs are enlarged, and the separated channels for DAPI and H5 staining are shown in grayscale. Scale bars: 10 μm.

Hyperphosphorylation of the Pol II CTD is considered a hallmark of 'active Pol II' that has progressed past the initiation stage and begun transcriptional elongation [3]. A monoclonal antibody H5, which recognizes the hyperphosphorylated form of the CTD repeat, is often used as a marker for this active Pol II status [6-8]. In *Drosophila *germline progenitor cells, recruitment of the P-TEFb kinase complex to promoter regions is directly inhibited by a small protein encoded by the *polar granule component *(*pgc*) gene. Pgc prevents phosphorylation of the CTD at Ser2, causing transcription to be stalled at a step before productive elongation [9]. A similar mechanism has been proposed in *Caenorhabditis elegans*, involving a maternal protein, PIE-1, which bears no sequence similarity to Pgc [9-11]. A transient cessation of transcription elongation has also been observed in mouse PGCs [12]. Global transcriptional repression is thus a conserved event in germ cell specification. Seydoux and Dunn reported that H5 staining is absent from the *C. elegans *P lineage (embryonic germline) blastomeres (P1 to P4), but appears in the two lineage-restricted PGCs, Z2 and Z3. PGCs are produced by the symmetric division of the last P blastomere (P4), and PIE-1 disappears from these cells shortly thereafter [13] (Figure 1A). Paradoxically, we have observed that a subset of conserved histone modifications associated with active gene expression disappear specifically from the PGC chromatin at or soon after this time [14,15].

We were intrigued by the simultaneous appearance and disappearance of epitopes that are all correlated with active transcription, and sought to investigate further the transcriptional regulation in the PGCs. We show that Pol II phosphorylation is uniquely regulated and transient in PGCs, suggesting that transcriptional repression is continued in the PGCs after the PIE-1 mode of repression is lost. This repression requires a component called maternal effect sterile (MES)-4, a histone H3K36 methyltransferase that is essential for fertility [16,17]. Genomewide chromatin immunoprecipitation followed by deep sequencing (ChIP-seq) analyses in embryos show that MES-4-mediated H3K36 methylation marks the bodies of genes that are expressed in germ cells, even those known to express only in post-embryonic stages. Our results are consistent with MES-4 contributing across generations to an epigenetic memory that marks germline-expressed genes and is required for normal germ cell development.

## Results

### Unique and dynamic regulation of Pol II status in the PGCs

**Figure 2:**
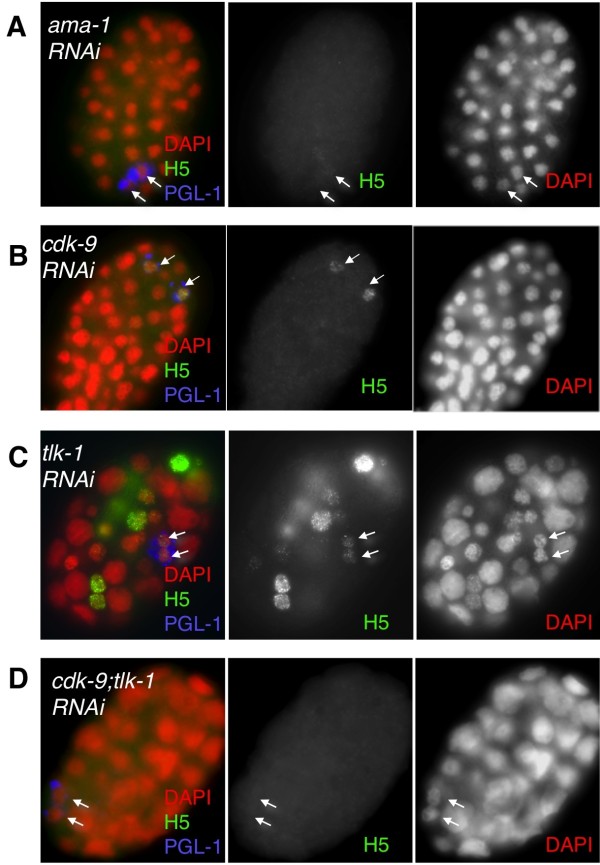
**Pol II CTD phosphorylation is uniquely regulated in primordial germ cells (PGCs)**. **(A) **H5 staining in *ama-1 (RNAi) *is depleted by *ama-1(RNAi)*. **(B) ***cdk-9 (RNAi) *embryo ablates H5 staining in all cells except the PGCs. **(C) ***tlk-1 (RNAi) *ablates H5 staining in many somatic cells, but the PGCs are often resistant. **(D) ***cdk-9; tlk-1(RNAi) *consistently ablates all H5 staining, including that in PGCs. Note that polyploid nuclei are observed in the *tlk-1 (RNAi) *embryo, as described previously [71]. H5 and DAPI staining (middle and right, respectively) are shown in grayscale with the merged image (left). Arrows indicate the PGCs.

We used the H5 monoclonal antibody, which recognizes the hyperphosphorylated form of Pol II (hereafter referred to as the H5 epitope), to investigate Pol II activity at different stages of PGC specification. We confirmed the specific absence of H5 staining in the P blastomeres and its appearance in Z2/Z3 soon after their birth [13] (Figures 1B, Figure 1C). To verify the specificity of the H5 staining, we also stained embryos exposed to RNA interference (RNAi) directed towards *ama-1 *(encoding the largest subunit of Pol II), *cdk-9 *(encoding a component of the predominant Ser2 kinase P-TEFb) [4] and *tlk-1 *(encoding Tousled-like kinase) [5] . In the *ama-1 *RNAi embryos, as expected, we observed loss of detectable H5 staining in all cells of embryos exhibiting the ~100-cell stage arrest characteristic of Pol II depletion (Figure 2A). Efficient depletion of the AMA-1 protein was verified in parallel samples (see Additional file 1). In *cdk-9 *RNAi embryos, we observed the same embryonic arrest phenotype as that in *ama-1 *RNAi with strongly reduced H5 staining in somatic cells, confirming previous observations (Figure 2B) [4]. Intriguingly, however, a substantial H5 signal was still observed in the PGCs in these embryos (Figure 2B, arrows). RNAi knockdown of *tlk-1 *also did not consistently ablate the H5 epitope in PGCs, although significant loss of H5 staining was observed in some somatic nuclei (Figure 2C). *tlk-1(RNAi) *embryos exhibited numerous other phenotypes, including cytokinetic defects [18], and we did observe some embryos lacking PGC H5 staining, so we cannot exclude some indirect effects on H5 staining in *tlk-1(RNAi) *embryos. However, full reduction of the H5 signal in PGCs was consistently observed with simultaneous knockdown of both *cdk-9 *and *tlk-1 *(Figure 2D). Note that the H5 signal was fully depleted in the PGCs of *ama-1 *RNAi embryos, indicating that in PGCs the H5 epitope that was resistant to *cdk-9 *and *tlk-1 *single RNAi is associated with AMA-1. These data suggest that Pol II CTD phosphorylation is regulated differently in PGCs than in the soma.

Remarkably, the H5 signal in wild type PGCs is transient. The strong H5 staining that initially appeared in PGCs soon after their birth (~200 minutes post-fertilization/~90 cells, at 22°C; Figure 1C) was not maintained in later stages and was significantly reduced by the 1.5-fold-stage (~450-500 minutes post-fertilization/~550 cells; Figure 1D). H5 staining remained very low in PGCs until hatching (~850 minutes post-fertilization) (Figure 1E). These observations suggest that Pol II may be transiently engaged in some stage of transcription elongation in the nascent PGCs, but that in later embryos the PGCs are largely transcriptionally inactive.

**Figure 3:**
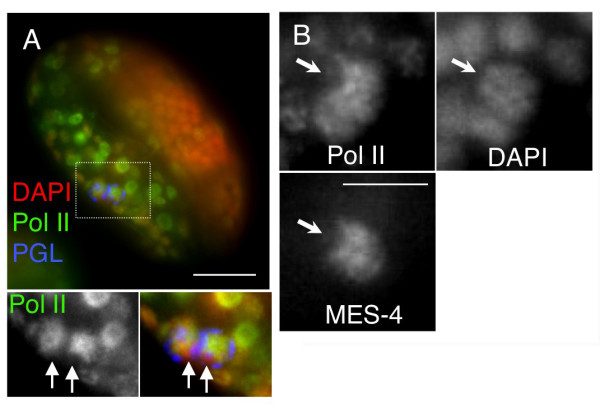
**A hypophosphorylated form of Pol II remains associated with the autosomes, but not the X chromosomes, in late primordial germ cells (PGCs)**. **(A) **Pol II is still on DNA late PGCs. DAPI, red; Pol II (8WG16 antibody) staining, green; PGL-1 staining, blue. Lower panels show enlarged PGCs and the separated channel for Pol II staining in grayscale. Arrows point to regions in the PGCs that lack Pol II staining. **(B) **The regions of Pol II exclusion overlap with regions lacking MES-4, which specifically associates with the autosomes at all stages that have been examined. Arrows point to the region of X chromosomes. Scale bars: **(A) **10 μm; **(B) **3 μm.

To determine if the loss of H5 staining in later stage PGCs also reflected loss of Pol II, we also stained the cells with a monoclonal antibody (8WG16) that can recognize the hypo-phosphorylated forms of Pol II [6,7,19]. As shown in Figure 3A, the 8WG16 signal was retained at high levels on PGC DNA long after the significant reduction in H5 staining. Interestingly, 8WG16 staining was consistently excluded from large, discreet regions of the PGC DNA Figure 3A, arrows), suggesting that the staining pattern was not a result of fixation-induced collapse of nucleoplasmic (soluble) Pol II onto DNA. We hypothesized that the regions excluding Pol II might be the X chromosomes, because the X chromosome is specifically silenced during larval and adult germ cell development, carries strikingly few germline-specific genes, and is thus a largely inert chromosome in most stages of germ cell development [20,21]. MES-4 protein is also excluded from the X chromosomes in the adult germline and in embryos [17]. The regions of PGC chromatin lacking Pol II also lacked MES-4, indicating that the regions are likely to be the X chromosomes (Figure 3B). Taken together, these observations suggest that in late PGCs, Pol II remains associated with chromatin in a hypophosphorylated and probably inactive state, and is preferentially associated with autosomes.

### The histone H3K36 methyltransferase MES-4 is required to maintain a repressed Pol II status in PGCs

Because histone modifications can be key elements in transcriptional regulation [3,22], we speculated that PGC-specific or enriched chromatin modifiers might be important for the peculiar dynamics of Pol II regulation observed in these cells. To test this possibility, we examined mutant strains of two known PGC-enriched histone modifiers, the H3K27 methyltransferase MES-2 and the H3K36 methyltransferase MES-4, both of which are essential for germ cell development [17,23]. MES-2, MES-3 and MES-6 assemble into a complex resembling the highly conserved polycomb repressive complex (PRC)2 [23-25]. MES-4 is a H3K36 methyltransferase, capable of both di- and tri-methylation, which is homologous to mouse NSD1 and can associate with chromatin independently of ongoing transcription [3] (see below, and [26]). Of the *mes *(*m*aternal *e*ffect *s*terile) mutants, *mes-4 *produces the most severe PGC proliferation defects [16,17]. Staining of these mutants revealed that H5 signal persisted at high levels in late-stage PGCs of *mes-4 *mutant embryos, compared with wild type controls (Figure 1D, Figure 1E, Figure 1H, Figure 1I; Table 1). This phenotype, and others detailed below, was observed in both the *bn67 *and *bn85 *alleles of *mes-4*, which encode a missense mutation in one of three PHD domains and a deletion in the SET domain, respectively [17]. By contrast, H5 staining was normal in *mes-2 *mutants (Figure 1J-L; Table 1). These data indicate that the H3K36 methyltransferase MES-4, but not the H3K27 methyltransferase MES-2, is important for the establishment and/or maintenance of transcriptional repression in late stage PGCs.

**Table 1:** Polymerase (Pol II) phosphorylation dynamics and H3K4me2 erasure in primordial germ cells (PGCs)

Genotype	H5 staining positive, % (n/total n)	H3K4me2 positive, % (n/total n)
Wild type (N2)	7 (4/54)	0 (0/37)

*mes-2 (bn11)*	9 (3/35)	3 (1/40)

*mes-4 (bn67)*	71 (32/45)	71 (15/21)
*mes-4 (bn85)*	65 (15/23)	78 (29/37)

Scoring results of late stage embryos with hyperphosphorylated Pol II (H5 staining) or H3K4me2-positive PGCs. Embryos at ~1.5-f to 2-fold stage were examined within a ~40 minute developmental window at around 500 minutes postfertilization at 20°C. A strict criterion was used to assess antibody signal: only those embryos with PGCs that showed comparable levels of H5 or anti-H3K4me2 staining relative to the staining in surrounding somatic cells were scored as 'positive'.

### MES-4 is required to maintain but not establish decreased H3K4me2 in PGCs

**Figure 4:**
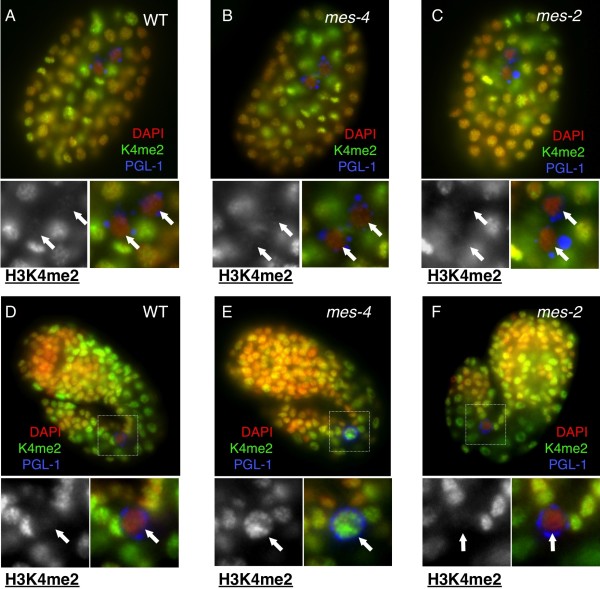
**H3K4 erasure in *mes-4 *primordial germ cells (PGCs) is normal, but exhibits precocious reappearance**. H3K4me2 staining in **(A-C) **early and **(B-D) **late stage primordial germ cells (PGCs of embryos from **(A,C) **wild type, **(B,D) ***mes-4(bn85) *and **(C,E) ***mes-2 *mothers. DAPI, red; H5, green; PGL-1, blue in merged images. In the lower panels, PGCs (boxed; arrows) are enlarged, and the H3K4me2 staining is shown in grayscale (left). Arrows mark PGCs.

Active transcription and histone H3K4 methylation can be coupled in many model systems [27,28]. This is also true for PGC chromatin, as *Drosophila *mutants of the P-TEFb recruitment inhibitor Pgc have premature activation of Pol II and precociously elevated H3K4me2 in the PGCs [9,29,30]. Given the persistence of active Pol II in *mes-4 *PGCs, we tested whether increased or precocious H3K4me2 is also present in *mes-4 *PGCs. As previously reported [14], we observed a normal erasure of H3K4me2 in both *mes-2 *and *mes-4 *PGCs soon after their birth (~200 minutes post-fertilization/~90 cells), as in wild type animals (Figures 4A-C). However, H3K4me2 levels substantially increased in later stage *mes-4 *PGCs (~500 minutes post-fertilization/~550 cells), in contrast to the near absence of H3K4me2 observed in wild type PGCs until the completion of embryogenesis (Figures 4D, Figure 4E; Table 1). By contrast, H3K4me2 dynamics in *mes-2 *PGCs were similar to those of wild type animals (Figure 4C, Figure 4F; Table 1). MES-4 is therefore not required for the H3K4me erasure mechanism in early PGCs, but is important for maintaining a repressive chromatin state in these cells. The reappearance of H3K4me2 that accompanies aberrant H5 staining in *mes-4 *PGCs suggests that H3K4me2 may be added to the chromatin as a consequence of aberrant transcription activation in *mes-4 *mutant PGCs. MES-4 is therefore a major contributor to transcriptional repression in wild type PGCs.

### MES-4 and methyltransferase (MET)-1 provide strikingly different modes of H3K36 methylation in the embryo

**Figure 5:**
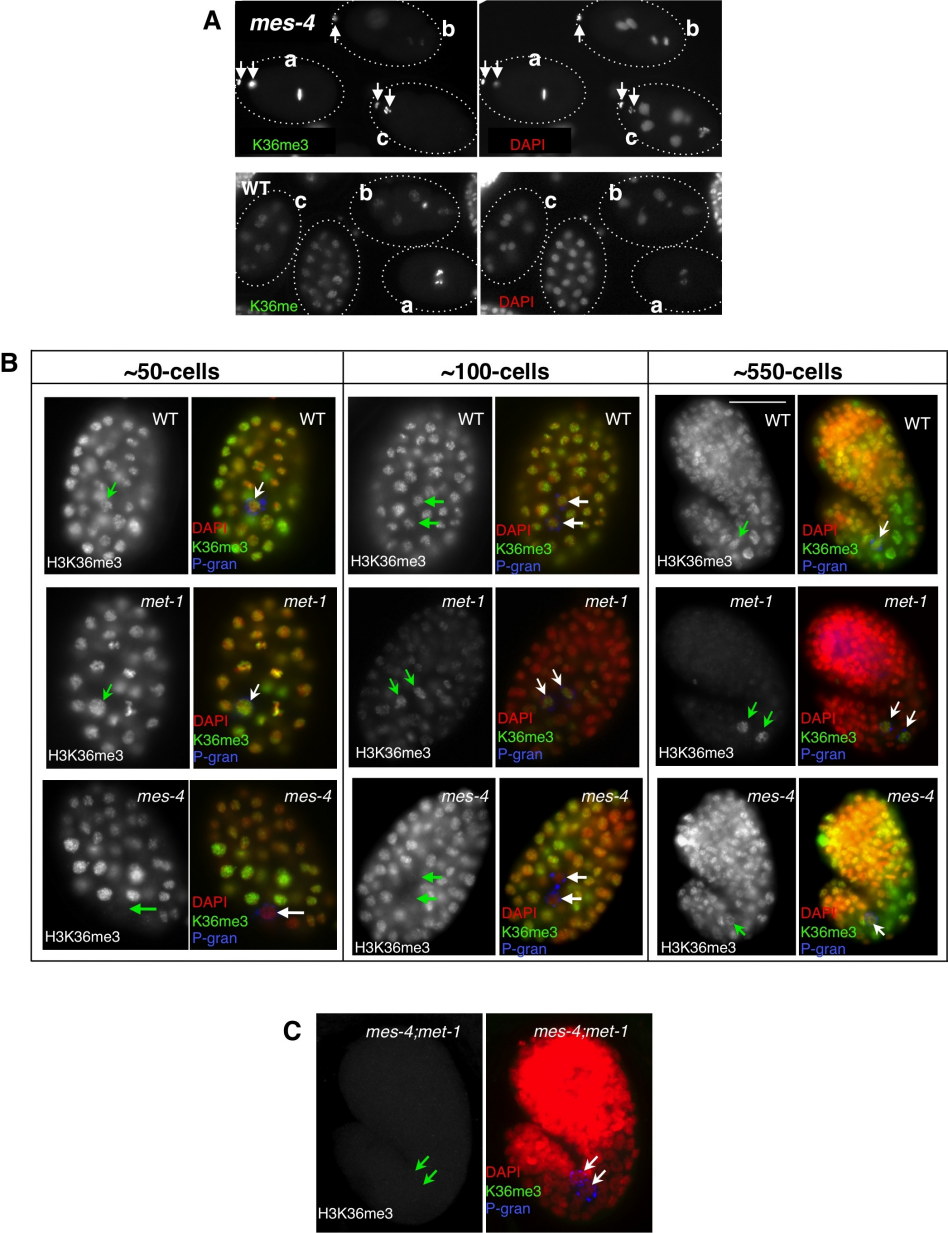
**H3K36 methylation dynamics during embryogenesis**. **(A) **MES-4 is required for the maintenance of H3K36me3 during early embryonic cell divisions. H3K36me3 (left) and DAPI staining (right) in *mes-4 (bn85) *M-Z- (top panels) and wild type (bottom panels) are shown in grayscale. Note that in *mes-4 *embryos, H3K36me3 that is prominently observed in dividing 1-cell stage embryo (embryo a), becomes barely detectable by the 2 to 4 cell stage (embryo b) and almost undetectable at the 6 to 8 cell stage (embryo c). Arrows indicate polar bodies. **(B) **H3K36me3 staining in wild type (top row), *met-1(n4337) *(middle row) and *mes-4(bn85) *(bottom row) at different embryonic stages (~50, ~100 and ~550 cells, as indicated on the top). DAPI, red; H5, green; PGL-1, blue in merged images. **(C) **H3K36me3 staining is not detectable in *mes-4(bn73)*; *met-1(n3347) *double mutant, Arrows indicate primordial germ cells (PGCs.

In many genomewide studies, H3K36 methylation has been correlated with actively transcribed genes, and indeed the yeast H3K36 histone methyltransferase (HMTase), Set2, cotranscriptionally modifies nucleosomes in the body of genes [22,28]. The *C. elegans *ortholog of Set2 is named MET-1 [31]. H3K36me3 is observed in all nuclei at all stages in wild type embryos (Figure 5A, bottom row; Figure 5B, top row). In *mes-4 *embryos, although H3K36me3 is initially observed in both pronuclei of the zygote, it is quickly lost from the chromatin after several cell divisions, presumably through replication-coupled histone dilution or replacement (Figure 5A). By around the 50-cell stage, significant H3K36me3 has become detectable in the somatic cells of the embryo; this methylation is consistent with MES-4-independent, MET-1-dependent HMT activity that is coupled with zygotic transcription activity [17]. However, *mes-4 *PGCs at this stage uniquely lack detectable H3K36me3 (Figure 5B, bottom), as has been previously reported for H3K36me2 [17]. The pattern in *met-1 *mutants, which carry only MES-4-dependent H3K36 methylation activity, is strikingly different. The H3K36me3 initially present in the parental pronuclei is maintained by MES-4 in early *met-1 *mutants (Figure 5B, middle row (and other data not shown)). Furthermore, the somatic maintenance of H3K36me3 progressively decreases but is stably maintained in the germline; indeed, the predominant signal in late stage embryos is in PGC chromatin (Figure 5B, middle row, arrows). This correlates with the previously reported late-stage enrichment of MES-4 in the PGCs [32]. No H3K36 methylation was observed in *met-1;mes-4 *double mutants at any stage, indicating that these factors are the sole providers of this modification in *C. elegans *(Figure 5C).

Interestingly, although *mes-4 *PGC chromatin initially lacked H3K36me3 (Figure 5B, middle of bottom row), this modification was observed to increase gradually in later stage PGC chromatin (Figure 5B, right of bottom row, arrow). This signal is dependent on MET-1, as it does not appear in the double mutant (Figure 5C, arrows). We cannot rule out that there is normally some MET-1-dependent H3K36 methylation in wild type late stage PGCs that is masked by the normal MES-4-dependent deposition of this marker. However, the H3K36 methylation appearing in late stage *mes-4 *PGCs coincides with the aberrant appearance of H3K4 methylation (Figure 4, Table 1), which suggests that the MET-1-dependent H3K36me3 is also aberrant. The aberrant presence in *mes-4 *PGCs of hyperphosphorylated Pol II, H3K4me and MET-1-dependent H3K36 methylation supports a conclusion that *mes-4 *mutant PGCs are precociously transcriptionally engaged. A summary of the dynamics for all of these modifications is shown (see Additional file 2).

We do not yet know if the precocious transcription that we observed in *mes-4 *PGCs represents premature activation of germline-expressed loci, ectopic activation of soma-expressed genes or some stochastic combination of both. We suspect that ectopic activation of genes expressed in soma is involved because (1) we often observed PGC chromatin structure changes that are reminiscent of somatic nuclei, such as a prominent nucleolus (Figure 1I; DAPI inset) and (2) unlike the absence of 8WG16 staining on the X chromosomes in late wild type PGCs, there is no obvious exclusion of H5 staining on any chromosome in late *mes-4 *PGCs (see Figure 3 vs. Figure 1I) (additional data not shown).

### MES-4-dependent H3K36 methylation can be uncoupled from transcription

The above results show that the genomewide maintenance of H3K36me3 in very early embryos, in both somatic blastomeres and the P-cells, is strictly dependent on MES-4 (Figure 5A). The somatic blastomeres have little requirement for genomewide transcriptional activity at these stages, because of the substantial maternal load of RNA and protein, and Pol II transcription is repressed in the P-cells [13]. Maintenance of H3K36me3 in these lineages despite little or no transcriptional activation suggests that MES-4 activity may be capable of operating independently of ongoing transcription. Indeed, previous studies have shown that MES-4-dependent H3K36 methylation in embryos is largely unaffected by RNAi knockdown of the Pol II large subunit AMA-1, whereas the MES-4-independent (MET-1-dependent) signal is strikingly decreased by *ama-1 *RNAi [17]. Genomewide immunoprecipitation plus microarray (ChIP-chip) analyses also indicate that MES-4 is enriched in gene that lacking detectable Pol II [26]. Thus, whereas MET-1 is required for H3K36me3 in transcriptionally engaged somatic nuclei, MES-4 can provide this modification in nuclei that are largely quiescent and/or those in which Pol II activity has been experimentally crippled.

**Figure 6:**
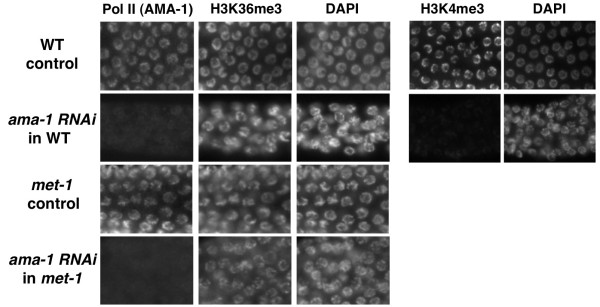
**H3K36 methylation by MES-4 is unaffected by Pol II knockdown in adult germ cells**. Wild type and *met-1(n4337) *animals soaked and fed *ama-1 *double-stranded (ds)RNAi for >50 hours and whole-mount fixed gonads were stained for Pol II (AMA-1; left columns) and H3K36me3 (left middle) or H3K4me3 (right middle). Comparable pachytene stage nuclei of indicated genotypes and treatment are shown. Note that both Pol II and H3K4me3 signals were significantly reduced *ama-1 *RNAi, whereas H3K36me3 was unaffected.

To test whether MES-4-dependent H3K36 methylation can also be transcription-independent in post-embryonic germ cells, we soaked *met-1 *L4 larvae in *ama-1 *double-stranded (ds)RNA for 24 hours and then fed these animals bacteria expressing the dsRNA. Adult *C. elegans *germ cells are organized into a spatial and temporal 'assembly line' within the gonad, with a stem cell population originating in the distal region progressing through meiosis and gametogenesis as the cells move into the more proximal regions. After ~50 hours of *ama-1(RNAi) *treatment, the gonads exhibited a distinct region of cells in the distal to mid-proximal region lacking detectable AMA-1 protein, representing germ cells born during the RNAi treatment. The chromatin in these nuclei also showed dramatically reduced H3K4me3 signal (Figure 6). By contrast, no decrease in MES-4-dependent H3K36me3 was apparent in the broad band of pachytene nuclei lacking detectable AMA-1 (Figure 6). Importantly, the bulk of adult germ cell transcription is thought to occur in the meiotic stages, where we observed efficient knockdown of both AMA-1 and H3K4me2 and retention of H3K36me3 in *met-1 *adults [33]. Active RNA Pol II is not detected on chromosomes at later stages such as in diakinetic oocytes [21].

The loss of both H3K4me3 and AMA-1 in the *ama-1(RNAi) *animals is evidence that both Pol II activity and its chromatin-associated consequences were strongly affected by the *ama-1 *RNAi treatment, yet this did not noticeably affect H3K36 methylation by MES-4 in the *met-1 *animals. This indicates that at least some MES-4-dependent H3K36me3 is uncoupled from ongoing transcription in all stages of the germline cycle tested. MET-1-dependent H3K36me3 signal was also present in adult germ nuclei of *mes-4 *M+Z- (homozygous null mutants produced from *mes-4*/+ heterozygous hermaphrodites). This was not observed in *mes-4;met-1 *double mutant germlines, indicating that MET-1 can provide H3K36 methylation in the adult germline, probably during ongoing germ cell transcription (data not shown). Interestingly, Bender *et al*. observed that H3K36me2 was not detectable in pachytene nuclei of *mes-4 *M+Z- adult germ cells [17], suggesting that MET-1-dependent H3K36 methylation is largely the trimethylated form in these cells.

### MES-4 is predominantly responsible for maintenance, but not *de novo*, H3K36 methylation in PGCs

Early embryos provide an informative window into the maternal to zygotic transition in control of K36 methylation. As illustrated in Figure 5A and described above, in *mes-4 *M-Z- embryos (lacking both maternal and zygotic MES-4 activity) H3K36me3 is clearly detectable in the pronuclear and early zygotic chromatin. This is presumably the result of MET-1 activity in the parental germline, but this signal is not maintained and disappears by the 4 to 8-cell stage (Figure 5A). The loss of this signal in *mes-4 *embryos suggests that MES-4 is either essential for maintaining pre-existing H3K36 methylation in embryonic chromatin through cell divisions or must provide this marker *de novo *after replication-coupled depletion.

**Figure 7:**
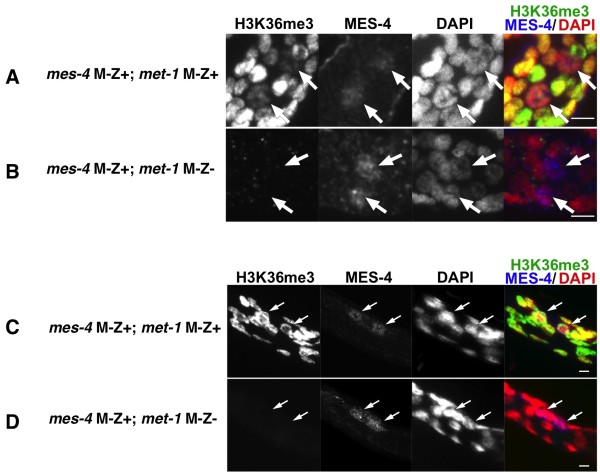
**MES-4 is unable to provide detectable *de novo *H3K36 methylation**. The maternal effect sterile (MES)-4 protein is expressed zygotically in the primordial germ cells (PGCs) of **(A,B) **embryos or **(C,D) **L1 larvae could not provide detectable H3K36me3 to animals that did not inherit maternal chromatin with H3K36me3. H3K36me3 was detectable in **(A) ***mes-4 *M-Z+;*met-1 *M-Z+ larval PGCs, but not in **(B) ***mes-4 *M-Z+;*met-1 *M-Z- PGCs. Similarly H3K36me3 was observed in **(C)**, *mes-4 *M-Z+;*met-1 *M-Z+ ~500 cell embryo PGCs but not in **(D) ***mes-4 *M-Z+;*met-1 *M-Z- PGCs. MES-4 indicates anti-MES-4 antibody staining. Arrows mark PGCs. Scale bars: 3 μm.

To test whether MES-4 has *de novo *H3K36 methylation activity, we examined zygotic MES-4 activity in *mes-4 *maternal null embryos (*mes-4 *M-Z+) in the absence of maternal MET-1 and in the presence or absence of zygotic MET-1 activity (*met-1 *M-Z- or M-Z+). Because of the cotranscriptional accumulation of H3K36me3 from zygotic MET-1 in somatic cells, we focused on PGCs to determine whether MES-4 could modify chromatin that lacked any pre-existing H3K36me3. Zygotically produced MES-4 protein was detectable in late stage PGCs (Figure 7A, Figure 7C). The presence of both zygotic MET-1 and MES-4 (in *mes-4 *M-Z+ animals) resulted in significant H3K36 methylation in the PGCs even in the absence of maternally premethylated H3K36 (Figure 7A). By contrast, MES-4 expressed zygotically in the PGCs could not detectably provide H3K36me3 on its own (that is, in the absence of maternally loaded MES-4 or MET-1) (Figure 7B). The same result was obtained in both early L1 larva stage germ cells (Figure 7C, Figure 7D) and in embryos (~500 minutes post-fertilization; Figure 7A, Figure 7B), both of which exhibited detectable, zygotically produced MES-4 protein. The zygotic MET-1-dependent H3K36me3 in the PGCs was detectable without maternal or zygotic MES-4 activity (Figure 5B). We interpret this to be a consequence of the precocious transcription in *mes-4 *PGCs described above. Regardless, it is clear that in the absence of prior or concurrent MET-1 activity, zygotic MES-4 cannot provide detectable H3K36 methylation on its own. These observations suggest that *in vivo*, MES-4 cannot provide H3K36 methylation *de novo*--that is, in chromatin lacking pre-existing H3K36me.

### MES-4 maintains H3K36me3 on germline-expressed loci in embryos, independently of transcriptional activity

We next wished to determine the genomic distribution of MES-4-dependent H3K36 methylation in embryos. H3K36 methylation mediated by Set2-type methyltransferases occurs as a consequence of ongoing transcription, so the distribution of this modification observed in mixed cell populations is likely to be dominated by its cotranscriptional addition. Indeed, the total amount of H3K36me3 detectable by western blot analysis in mixed-stage *met-1 *embryos is reduced by ~90% compared with wild type levels [31]. To observe only MES-4-dependent methylation, we examined H3K36me3 patterns at high resolution in *met-1 *mutant embryos using ChIP-seq. In contrast to *mes-4 *mutants, which exhibit maternal effect sterility, the *met-1(n4437) *deletion strain is homozygous viable and fertile [31]. We isolated mid to late stage *met-1 *embryos, which are transcriptionally fully active and within which somatic developmental pathways should be fully engaged. Although both MES-4 protein and MES-4-dependent H3K36me3 become enriched in PGCs at late stages (Figure 5B), the embryo samples used for both *met-1 *and wild type ChIP-seq contained a mixture of embryos with significant somatic levels of both MES-4 protein and its histone marker (see Additional file 3). We therefore believe that the vast majority of H3K36me3 material we obtained by this method is from the 300-500 somatic nuclei, rather than the two PGC nuclei, in these embryos.

**Figure 8:**
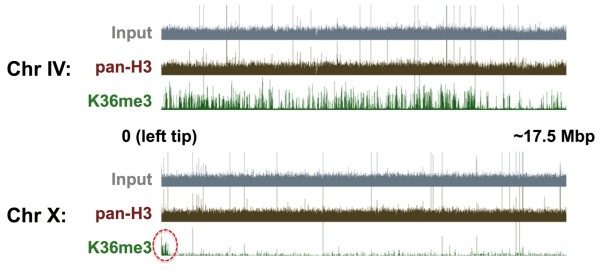
**H3K36me3 ChIP-seq in *met-1 *embryos detects maternal effect sterile (MES)-4-mediated histone methylation**. MES-4-mediated H3K36me3 (green), pan-histone H3 (brown) and input (gray) profiles on an autosome (chromosome IV) and the X chromosome in *met-1 *mutant late embryos. The red circle indicates MES-4-mediated H3K36me3 signal on the left tip of the X chromosome. The signals for pan-H3 and input are partly magnified relative to that of H3K36me3. Averages from two independent experiments are shown.

In both wild type and *met-1 *embryos, the MES-4-mediated H3K36me3 ChIP-seq signal was significantly lower on the X chromosome compared with the autosomes, and the signal on the X was concentrated in the left tip (Figure 8). This is consistent with the reported distribution of MES-4 protein by immunofluorescence and recent ChIP-chip results for MES-4 protein [17,26]. By contrast, pan-H3 control ChIP and input samples did not show significant differences in signal levels between the X chromosome and autosomes. These results indicate that ChIP-seq in *met-1 *mutants appropriately enriches for MES-4-mediated H3K36me3 signals.

**Figure 9:**
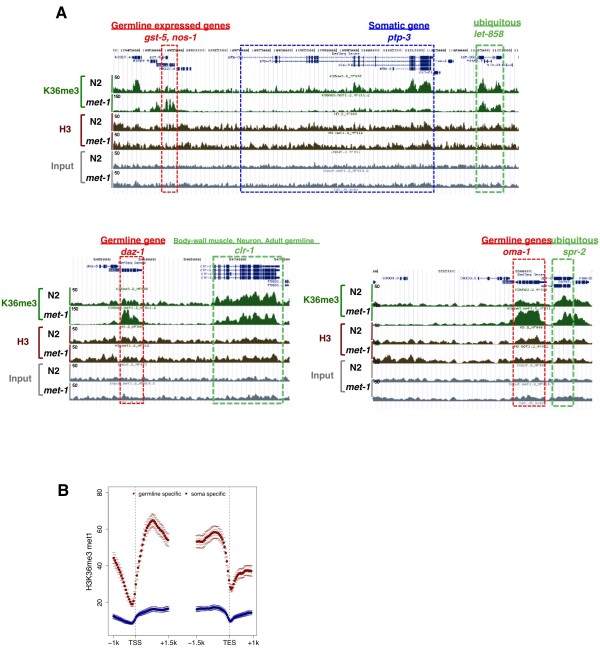
**H3K36 methylation by maternal effect sterile (MES)-4 is enriched in transcribed regions of germline expressed loci**. **(A) **Screen shots of H3K36me3 (green), panH3 (brown) and input patterns (gray) in wild type and *met-1 *background mapped onto the UCSC genome browser for several regions are shown together with the annotated gene models (blue, top of each panel). (Top) MES-4-mediated H3K36me3 was enriched on germline-expressed genes, *gst-1 *and *nos-1*(red box), but not on a somatic gene *ptp-3 *(blue box), compared with the methylation pattern in wild type, whereas the methylation pattern on a ubiquitously expressed gene *let-858 *(green box) was similar in both wild type and *met-1 *backgrounds. (Bottom left): *daz-1*, which is essential for meiotic progression during oogenesis and is expressed specifically in the larval/adult germline [72] is a locus at which MES-4-mediated methylation is significantly enriched. (Bottom right): Another example of specific enrichment of MES-4-mediated H3K36me3 on the adult germline-specific gene *oma-1*, which is required for oocyte maturation. The *y*-axis is the signal intensity (the number of illumina GA sequence reads). **(B) **Gene profiles showing MES-4-mediated H3K36me3 on larval/adult germline-specific genes (red; see also Figure 10E) and soma-expressed genes (blue; see also Figure 10D) in *met-1 *embryos. Values are average scores for 50 bp intervals in the 2.5 kb surrounding the transcription start site (TSS) and the transcription end site (TES). Error bars indicate the 95% confidence interval.

We next identified where H3K36me3 was enriched within *met-1 *late embryonic chromatin by mapping the sequence reads to the *C. elegans *genome (WormBase version WS170, ce4) using MAQ software http://www.maq.sourceforge.net. H3K36me3 was enriched within the coding region of annotated genes in both *met-1 *and wild type (N2) chromatin (Figure 9). Interestingly, initial analyses of the H3K36me3 patterns in the *met-1 *sample appeared to show a relative enrichment in germline-expressed loci, but not in genes annotated or predicted to be expressed in somatic lineages. We verified this pattern in *met-1 *embryos for a number of genes by quantitative (q)PCR (see Additional file 4). This relative difference was not observed in the wild type N2 experiments (Figure 9A).

**Figure 10:**
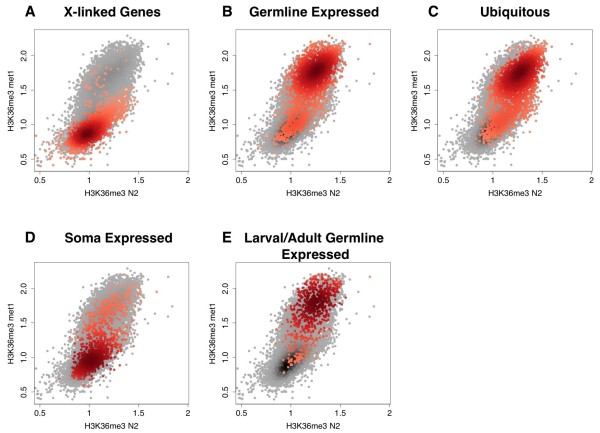
**The maternal effect sterile (MES)-4 protein marks genes expressed in germ cells but not soma**. **(A-E) **Scatter plot comparison between H3K36me3 in *met-1 *(mediated by MES-4) and H3K36me3 in wild type (mediated by MES-4 and methyltransferase (MET)-1). Gray dots show the log_10 _average read counts for base pairs between the transcription start and end site for all genes. Red dots highlight **(A) **X-linked genes, **(B) **germline-enriched genes, **(C) **ubiquitously expressed genes, **(D) **soma-expressed genes and **(E) **larval/adult germline-specific genes. See text and supplemental experimental procedures for descriptions of gene sets.

We further analyzed this for the entire genome. The number of reads for any locus exhibiting relative enrichment for H3K36me3 in both N2 and *met-1 *samples was lower in N2 than in *met-1*, on average, but the enrichment for H3K36me3 in annotated genes was readily observed in all samples. This allowed us to determine which genes were enriched for H3K36me3 within each sample, and then compare these gene sets between wild type and *met-1 *experiments (see Additional file 5). Scatter plot analyses of H3K36me3 levels on different gene sets in *met-1 *and N2 showed high levels of MES-4-mediated H3K36me3 levels on germline-expressed genes in *met-1*, including those genes expressed specifically in the germline (see below) and genes generally expressed in all cell types (Figure 10B, Figure 10C, Figure 10E). In striking contrast, genes expressed in the somatic lineages of embryos showed little or no MES-4-mediated H3K36me3 in *met-1 *(Figure 10D).

Interestingly, a significant number of genes annotated as having somatic expression also showed H3K36me3 enrichment in the *met-1 *experiment (Figure 10D). We further analyzed the expression pattern of these genes in a public *in situ *database (NEXTDB; http://nematode.lab.nig.ac.jp/. Of those genes with a discernable expression pattern, almost all (17/18) showed expression in adult germ cells, indicating that either these genes may be ubiquitously expressed or that their somatic designation is in error (not shown). Collectively, these data suggest that MES-4 maintenance of H3K36me3 in *met-1 *embryos is largely, if not solely, devoted to genes whose expression occurs in postembryonic larval and adult germ cells. This is again consistent with results obtained by MES-4 ChIP-chip [26].

Genes that are specifically transcribed in the larval/adult germline but not during embryogenesis would not exhibit transcription-dependent H3K36me3 in embryonic chromatin. We therefore determined if these genes are still marked by MES-4 in *met-1 *embryos. We selected the intersection of germline-expressed genes (that is, data published in [34,35]) and 'strictly maternal' genes identified in *C. elegans *embryonic transcriptome analyses (see experimental procedures). Scatter plot analysis using the 'larval/adult germline-specific' gene set showed obvious enrichment of MES-4-mediated H3K36me3 on most of these genes (Figure 10E). Profiling of H3K36me3 signals across the transcription start and transcription end sites revealed that MES-4-mediated H3K36me3 is enriched in the bodies of these genes, whereas MES-4-mediated H3K36me3 ChIP-seq reads are detected at very low frequencies in the bodies of genes known to be expressed in somatic cells in the embryonic stages examined (Figure 9B). Interestingly, the MES-4-dependent pattern of H3K36 methylation in germline-expressed loci shows a slight enrichment at the 5' end, whereas transcription-coupled H3K36me3 is usually more enriched toward the 3' end of gene bodies [36]. These results indicate that MES-4 activity in *met-1 *embryos is maintaining H3K36 methylation within the transcribed portion of germline-expressed genes, even though transcription of these genes last occurred in the adult germ cells of the previous generation.

## Discussion

In this study, we show that Pol II phosphorylation and transcriptional activity is uniquely regulated in the primordial germ cells of *C. elegans*, and that this regulation requires the H3K36-specific methyltransferase MES-4. The unique retention of Pol II phosphorylation in the PGCs that we observe with *cdk-9(RNAi) *indicates that Pol II activity is regulated differently in these cells. Indeed, the transience of this phosphorylation state followed by a prolonged association of the hypophosphorylated form with only autosomal chromatin is clearly unusual. This autosomal restriction is probably related to the paucity of germline-expressed loci on the X chromosome, and may indicate that Pol II is 'poised' at such loci, held ready at the gate in advance of larval germ cell activation at hatching. The requirement for MES-4 to maintain this status suggests that H3K36 methylation contributes to this germ cell-specific form of Pol II regulation.

H3K36 methylation is intriguing because although its addition has been generally correlated with actively transcribed genes, it has been shown also to negatively affect transcription when ectopically recruited to promoters, and can prevent initiation at cryptic promoters [37]. Interestingly, this dichotomy has been reported to depend on the local H3K4 methylation state. In budding yeast, histone acetylation is promoted by coincidence of H3K36 and H3K4 methylation [38-40]. H3K36 methylation can recruit the Rpd3 S HDAC complex and repress initiation from cryptic intragenic promoters, and this occurs most often in the 3' regions of gene bodies, where Set2-dependent H3K36me is high and Set1-dependent H3K4me is low [41-45]. In *C. elegans*, H3K4 methylation is extensively erased from the chromatin in PGCs shortly after they are born [14]. By contrast, H3K36me3 is actively maintained by MES-4 in these cells. Assuming the pattern of MES-4 dependent H3K36me3 is consistent in the PGCs, this would create a chromatin status in the 5' region of germline-expressed genes similar to that described above for gene bodies in yeast-- that is, enriched in H3K36me3 and low in H3K4me (Figure 1A, Figure 9B). It is thus possible that the presence of MES-4-mediated H3K36 methylation combined with an absence of H3K4me may present a repressive chromatin signature that prevents or suppresses sustained Pol II activation at most genes in the PGCs. When the PGCs activate after hatching and H3K4 methylation returns, this repressive combination no longer exists and active germline transcription ensues, perhaps through reactivation of Pol II already present (poised) at these loci.

Importantly, the defective PGC repression we observed in *mes-4 *PGCs was independent of the allele used. The *bn67 *allele is a point mutant converting a histidine to tyrosine in one of the three PHD domains of MES-4, whereas the *bn85 *allele is a deletion that disrupts the SET domain. Neither mutation creates a protein null mutant, but both abrogate the localization of MES-4 to DNA [17]. It is thus possible that MES-4 activities other than addition of H3K36me2/3 to histones are required for Pol II repression in the PGCs, and that these activities require recruitment to the DNA for repression. However, the PHD and SET domains (including AWS-like, SET and post-SET motifs) are the only recognizable domains of MES-4: both are known to interact with histones and both are required for H3K36 methylation by MES-4. A direct role of H3K36 methylation in Pol II repression in the PGCs awaits future analysis using a *mes-4 *methyltransferase catalytic mutant.

MES-4 appears to 'maintain' rather than establish H3K36 methylation in genes, independently of their transcriptional status. We base this model on the following observations: (1) In the absence of MES-4, the H3K36me3 arriving in gamete chromatin is quickly diluted by replication and cell division (Figure 5A); (2) zygotically provided MES-4 cannot contribute detectable H3K36 methylation on its own in larval or embryonic germ cells (Figure 7); (3) neither normal transcriptional quiescence (for example, P cells) nor experimental disruption of Pol II activity in germ cells noticeably affects H3K36 methylation (Figure 5, Figure 6); (4) in embryos, MES-4-dependent H3K36me3 is enriched within the body of genes that are known to be expressed only in post-embryonic germ cells (Figure 9); (5) the gene-body distribution shows an unusual 5' enrichment, which does not overlap with the reported distributions of either H3K36me3 or Pol II in other organisms and, (6) also in embryos, MES-4-dependent H3K36me3 is not enriched within the body of somatically-expressed genes that are transcriptionally active in the stages analyzed (Figure 9, Figure 10). A recent ChIP-chip analysis of the genomewide distribution of MES-4 in embryos confirms that MES-4 is enriched in germline-expressed loci and that this enrichment is independent of Pol II occupancy [26].

There has been considerable controversy regarding the ability of histone modifications to provide stable and heritable epigenetic information, given the extensive nucleosome dynamics throughout the cell cycle. However, recent studies have identified molecular mechanisms that allow epigenetic markers in both DNA and chromatin to be actively maintained despite such dynamics. The maintenance of DNA methylation is mediated by recognition of hemimethylated CpG sequences by the SRA domain of UHRF1, which recruits Dnmt1 to the DNA [46-50]. Similarly, the PRC2 complex, which catalyzes H3K27me3, was shown to bind to pre-existing H3K27me3, and this binding appears to be crucial for the maintenance of this modification in proliferating cells [51,52]. MES-4 may be similarly stabilized on nucleosomes through its three PHD domains, which have been shown to help recruit the Rpd3 S complex to nucleosomes marked by methylated H3K36 in yeast [44]. Interestingly, both the *mes-4 bn67 *allele studied here and another allele with mutations in one of the PHD domains (*bn50*) cause dissociation of MES-4 from chromatin and the same germ cell degeneration phenotype observed in null alleles [17]. Mutations in *mrg-1*, which encodes a germline-enriched chromodomain protein, cause PGC proliferation defects similar to those in *mes-4 *mutants and misregulation of genes that are also misregulated in *mes-4 *mutants. The MRG-1 protein, like MES-4, is also excluded from the X chromosome [53]. MRG-1 may thus also participate in PGC transcriptional repression by MES-4.

The maintenance of epigenetic information is particularly relevant to transmission through the germline, as this information has the potential to affect gene regulation across multiple generations. The maintenance of H3K36me by MES-4 in embryos, which we observed in the bodies of genes transcribed in adult germ cells, suggests that this marker is important for such gene regulation. Indeed, a transgenerational requirement for this marker is revealed by the *mes-4 *(maternal effect sterile) phenotype; the dysregulation that occurs in *mes-4 *PGCs is not observed until a full generation after the actual loss of MES-4 protein activity. In *mes-4 *M+Z- embryos, the maternally provided MES-4 protein becomes focused in the PGCs as in wild type embryos and maintains H3K36 methylation status in these cells. When the embryo hatches and germline development progresses, the thousand or so germ cells arising from these PGCs are functional and can develop into normal gametes, despite having only transcription/MET-1-dependent H3K36 methylation. However, in the next (M-Z-) generation, the MET-1-dependent H3K36me3 arriving within the gamete chromatin is not maintained and the 'information' is lost in the embryonic germline. The PGCs lose stable Pol II repression, and their few descendents degenerate after postembryonic activation [16]. MES-4 is thus important for maintaining the H3K36m3 marker in germline genes, which may be essential for their normal regulation in the germ cells of subsequent generations.

The proposed maintenance histone methylation activity can also potentially explain, at least in part, the strict maternal effect sterility observed for *mes-4 *mutants, in which zygotically supplied MES-4 cannot rescue the germ cell degeneration phenotype (for example, M-Z+ embryos). The absence of maternal MES-4 maintenance activity in the embryonic germ cells would result in the loss of inherited H3K36me template through replication-coupled dilution (Figure 5A), yielding little 'template'/substrate for the zygotic MES-4 activity, which is not synthesized until later in development (~300-400 cells) (H. Furuhashi, unpublished results). Therefore, no rescue by zygotic MES-4 is observed.

Curiously, the marking of germline-expressed genes by MES-4 is also crucial for allowing ectopic activation of germline-expressed loci in somatic cells of mutant backgrounds that are defective in global transcriptional repression mechanisms [54,55]. The reasons for this ectopic expression and for the requirement for MES-4 activity to allow the expression to occur in post-embryonic stages are currently unknown. The requirement for MES-4 marking to yield ectopic germline gene expression when somatic modes of repression are defective may indicate that H3K36 methylation plays a prominent role in marking genes for default expression when somatic repression mechanisms are absent (as in germ cells) or defective (as in the mutants).

The noted lack of association of MES-4 with the X chromosome in embryos can be readily explained by the paucity of germline-expressed loci on the X chromosome [20]. The methylation pattern of MES-4 in the embryo may represent the maintenance of H3K36me3 at loci that were originally marked by transcription-coupled H3K36 methylation in the adult germ cells of each preceding generation. The only genes within which such 'transcriptional memory' could be transmitted across generations would be those that are expressed in adult germ cells--that is, the pattern we observed in *met-1 *embryos. An intriguing possibility is that the pattern of H3K36 methylation in *met-1 *embryos is evidence that this memory can be highly stable-that is, that loci originally marked by transcription-coupled MET-1 activity when present in the strain many, many generations previously is still being faithfully maintained by MES-4. The gene-body distribution of MES-4-dependent H3K36me3 in embryos indicates that it can be concentrated in areas where germ cell transcription occurred in the parental germline, yet where there is no evidence of ongoing transcription in the embryo. Indeed, a recent ChIP-chip analysis of MES-4 protein in wild type embryos showed that MES-4 protein is found within germline-expressed loci that lack detectable Pol II [26]. However, although we have strong evidence that MES-4 activity can operate independently of transcription, we cannot know with certainty that this is always the case. Nevertheless, it is clear that MES-4 can provide stable maintenance across generations of H3K36me in germline genes, regardless of their transcriptional status.

MES-4 appears to be a metazoan-specific H3K36 HMTase. MES-4-related proteins in other systems (dMES-4 in *Drosophila *and the NSD family of proteins in mammals) are crucial for normal development and are implicated in various cancers and developmental disorders such as Sotos and Wolf-Hirschhorn syndromes [56-59]. The molecular mechanisms underlying the developmental requirement for these proteins in these organisms are not clear. Indeed, the role of MES-4 in *C. elegans *is somewhat paradoxical. For instance, although MES-4 is largely absent from the X chromosome, genes on the X chromosome are the major targets of dysregulation in *mes-4 *(M+Z-) mutant adult germ cells [17]. It has been proposed that MES-4 indirectly affects X chromatin structure by preventing repressive factors from accessing autosomal chromatin, thereby focusing their action on the X chromosome [17]. In *mes-2/3/6 *mutants, MES-4 is observed to localize ectopically to the X chromosome in oocytes [32]. This apparent connection between MES-4 and the other MES complex in adult germ cells may be separable from PGC-specific processes, because *mes-2 *mutations do not affect the PGC processes we studied.

## Conclusions

Our results indicate that H3K36 methylation can serve as an important component of epigenetic memory, and that this memory is required for germline continuity in *C. elegans*. Although H3K36 methylation has been generally correlated with ongoing transcription elongation [22,28], the H3K36me3 that is enriched in the *C. elegans *embryonic germline chromatin is dependent on MES-4, a methyltransferase whose activity can be independent of Pol II, as detailed above [17,26]. H3K36 methylation on germline-expressed genes is stably maintained by MES-4 across generations, and our data suggest that MES-4 and/or the information transmitted by its histone modification product play a key role in preventing abnormal transcriptional activation in the PGCs of subsequent generations. These results provide new insights and identify additional modes of chromatin-based, transgenerational transcriptional regulation in metazoan development and germline specification.

## Methods

### Worm strains

*C. elegans *N2 Bristol strain was used as the wild type. The following mutations, balancers and translocations were used: LGI: *met-1(n4337)*; LGII: *mes-2(bn11), unc-4(e120), mnC1; *LGIV: *DnT1[qIs51](IV;V); *LGV: *dpy-11(e224), mes-4(bn67, bn73, bn85), DnT1[qIs51](IV;V)*

*met-1(n4337) *is a deletion mutant that lacks the SET domain [31]. *mes-2*(*bn11*) is a point mutation that results in a premature stop codon before the SET domain; it produces no detectable protein and very little to no H3K27me3 staining in the PGCs [23,60]. *mes-4(bn85) *has an inframe deletion that disrupts the SET domain. *mes-4(bn73) *has a premature stop codon after amino acid 593 in the middle of the SET domain. *mes-4(bn67) *has a point mutation in its first PHD finger, which leads to complete dissociation of MES-4 protein from chromosomes [17]. *unc-4(e120) *and *dpy-11(e224) *are visible genetic markers.

### RNAi analysis

RNAi was performed to deplete RNA Pol II/AMA-1, CDK-9 and TLK-1 from embryos by microinjection into parent worms as described previously [4]. Embryos in the injected animals were dissected out after ~24 h at 20°C, and prepared for whole-mount fixation and immunofluorescence analyses (see below). To deplete Pol II/AMA-1 from adult germlines, L4 larvae were soaked in 0.5 μg/μl *ama-1 *dsRNA for 24 hours at 20°C. The animals were then transferred onto NGM/Amp/IPTG plates (3g NaCl + 17g agar + 2.5g Bacto-peptone in 1L dH_2_0, made 50 μg/ml ampicillin and 1 mM IPTG) seeded with HT-115 bacteria that had been transformed with an *ama-1 *dsRNA expression plasmid or control L4440 plasmid. The feeding RNAi was performed at 20°C for ~55 hours, during which the feeding plate was exchanged once after the first 24 hours.

### Immunofluorescence staining

Samples were fixed using methanol/formaldehyde [13] for H5 staining, and methanol/acetone [14] for H3K4me2 and H3K36me3 staining. For 8WG16 and MES-4 staining, embryos were fixed in 2.5% paraformaldehyde for 2 minutes, followed by a 2 minute post-fix in -20°C methanol. Primary antibodies used were: affinity-purified rabbit anti-MES-4 [17] (1:10), mouse monoclonal CMA333 to H3K36me3 (0.25 μg/ml, rabbit anti-PGL-1 [61] (1:10,000), mouse monoclonal OIC1D4 to P-granules [62] (1:4), mouse monoclonal H5 to hyper-phosphorylated Pol II CTD (1:50) [6] and mouse monoclonal 8WG16 to Pol II (Covance) (1:100). We extensively characterized the specificity of monoclonal H3K36me3 (CMA333) by immunostaining (Figure 3; see Additional file 3), immunoblotting and ELISA (data not shown). For H3K4me2 staining, we used both a fully characterized monoclonal antibody CMA303 [63] and a rabbit polyclonal antibody (Millipore Corp., Billerica, MA, USA) Both antibodies yielded essentially the same results for the staining of PGC nuclei, although the polyclonal antibody tended to produce slightly higher background staining (data not shown). The data shown were obtained using the monoclonal anti-H3K4me2 antibody. Secondary antibodies used were Alexa Fluor 488-conjugated goat anti-mouse and Alexa Fluor 594-conjugated anti-rabbit antibodies (1:500) (Molecular Probes, Eugene, OR, USA). Samples were mounted in anti-fade reagent (ProLong Gold; Molecular Probes) and observed under a fluorescence microscope (Leica DMRXA; Hamamatsu Photonics, Hamamatsu, Japan) with Simple PCI software (Hamamatsu Photonics).

### ChIP-sequencing analysis

Synchronized, gravid young *met-1 *or wild type N2 adults were rinsed with M9 buffer and lysed in freshly prepared egg isolation solution (20% fresh commercial bleach, 500 mM NaOH) to collect embryos. Isolated embryos were rinsed with M9 buffer and separated from lysed worms and other debris by sucrose flotation as described previously [64]. To allow embryos to develop to later stages, collected embryos were incubated in M9 buffer at 20°C for 5 hours. An image showing a representative field of embryos after isolation is provided (see Additional file 3). After incubation, eggshells were digested by treatment with egg buffer (25 mM HEPES pH7.4, 118 mM NaCl, 48 mM KCl, 2 mM MgSO_4_, 2 mM CaCl_2_) containing 1U/ml of chitinase (C6137; Sigma Chemical Co., Sigma, St Louis, MO, USA) at 20°C for 40 minutes. The embryos were rinsed with egg buffer and frozen in liquid nitrogen. The frozen embryo pellet (100-150 ul vol) was resuspended in 1 ml phosphate-buffered saline (PBS) containing protease inhibitor cocktail (Roche Applied Sciences, Indianapolis, IN, USA) and homogenized with a Dounce homogenizer. To fix chromatin, formaldehyde was added to the homogenate to a final concentration of 1%, and the tube rocked for 10 minutes at 25°C. After quenching with 125 mM glycine, the nuclei/chromatin fraction was collected by centrifugation at 900*g *for 1 minute, washed with 1ml PBS three times, and resuspended in 400 μl SDS lysis buffer (included in ChIP Assay Kit; Millipore Corp., Billerica, MA, USA) containing protease inhibitor cocktail. The fixed material was placed in a sonicator to give sheared chromatin preparations with an average DNA size of ~300 bp. After centrifugation at 13,000*g *for 10 minutes, the supernatant was collected and diluted 10-fold in ChIP dilution buffer (16.7 mM Tris-HCl pH7.5, 167 mM NaCl, 0.01% SDS, 1.1% Triton X-100, 1.2 mM EDTA). The DNA concentration of the chromatin preparations was determined and adjusted with LSW buffer (20 mM Tris-HCl pH7.5, 1 mM EDTA, 1% Triton X-100, 0.1% SDS, 150 mM NaCl) to ~20 μg/ml for H3K36me3 ChIP in *met-1*, ~10 μg/ml for H3K36me3 ChIP and ~5 μg/ml for pan-H3 ChIP in both genotypes. Aliquots of 2 ml (for H3K36 ChIP) or 1 ml (for pan-H3 ChIP) of chromatin preparation were mixed with 5 μg of monoclonal anti-H3K36me3 (CMA333 (supplied by co-author, H. Kimura)) or 2.5 μg of monoclonal anti-H3 (ab10799; Abcam Inc., Cambridge, MA, USA) overnight at 4°C on a rotator. Magnetic beads (150 μl) coated with anti-mouse IgG (Dynabeads; Dynal Bead Based Separations (Invitrogen Group) Carlsbad, CA, USA) were then added to each 1 ml reaction and rotated for 3 hours at 4°C. Beads were washed for 3 minutes with 1 ml of each of the following buffers in succession: LSW buffer, HSW buffer (20 mM Tris-HCl pH7.5, 1 mM EDTA, 1% Triton X-100, 0.1% SDS, 500 mM NaCl) and TEL buffer (0.25 M LiCl, 1% NP-40, 1% sodium deoxycholate, 1 mM EDTA, 10 mM Tris-HCl pH8.1), followed by two final washes with TE buffer (10 mM Tris-HCl pH8.0, 1 mM EDTA). Samples were eluted twice with elution buffer (10 mM Tris-HCl pH8.0, 1 mM EDTA, 1% SDS, 250 mM NaCl) for 15 minutes at 65°C, treated with proteinase K at 55°C for 1-2 hours, then transferred to 65°C overnight to reverse-crosslink. DNA was purified with a commercial kit (PCR Purification Kit; Qiagen, Valencia, CA, USA) and eluted in 40 μl 10 mM Tris-HCl pH 8.0. Eluate DNA was quantified (PicoGreen; Invitrogen) and adjusted to 1 ng/μl.

Libraries were prepared from 30 ng of purified immunoprecipitated DNA or input DNA and analyzed on a genome analyzer (Illumina Inc., San Diego, CA, USA) at the UNC sequencing facility. Experiments were carried out using two biologically independent samples.

The sequenced reads were mapped to the *C. elegans *genome (WormBase version WS170, ce4) using MAQ http://www.maq.sourceforge.net using default parameters. The mapped 36 bp reads were extended to 200 bp. For each base pair in the genome, the number of overlapping sequence reads was determined. For all replicates of Input and H3 and H3K36me3 ChIP experiments, the read count per base pair was scaled so that the median read count across the genome was the same. Read counts per base pair were averaged across replicates and visualized in the UCSC genome browser http://genome.ucsc.edu/.

Both met-1 ChIP-seq experiments displayed robust H3K36me3 signal over numerous open reading frames with significantly lower signal in most non-coding regions; we consider the latter to be 'background' or 'noise'. Both individual N2 ChIP-seq experiments had a somewhat lower signal to background ratio. Despite the difference in signal to background and the fact that read counts per base pair are not directly comparable between *met-1 *and N2, we could readily identify a large number of genes with clear enrichment of H3K36me3 in both samples. This allowed us to compare H3K36me3 on genes in particular categories (for example, those expressed specifically in germ cells or expressed in somatic cells) between the two genotypes.

Scatter plots (Figure 10) were generated by averaging the read counts of base pairs within the transcription start and end sites for each gene. Log_10 _of the average read counts per gene were plotted for H3K36me3 in *met-1 *versus N2.

Average profiles of H3K36me3 in *met-1 *around transcription start sites (TSS) and transcription end sites (TES) for larval/adult germline-specific and soma-expressed genes are shown in Figure 10. Read counts per base pair were averaged in 50 bp intervals for each gene from 1 kb upstream to 1.5 kb downstream of the TSS and 1.5 kb upstream to 1 kb downstream of the TES. Genes were aligned at the TSS and TES, and the average read count calculated for germline-specific and soma-expressed genes in 50 bp steps. Error bars indicate the 95% confidence interval for the mean of each 50 bp interval.

Custom perl scripts and Bioconductor packages for R http://www.r-project.org; http://www.bioconductor.org were used for analysis of the data and generation of the figures.

### Gene set definitions

The gene sets (Figure 9) were defined based on various expression data sets. In total, 2243 germline-enriched genes were obtained from previous work [34]; spermatogenesis genes were excluded from this set. Ubiquitously expressed genes (n = 2580) were defined as genes expressed in germline, muscle, nerve and gut, according to SAGE analysis (SAGE tag count ≥ 1) [35,65]. Somatic genes (n = 1273) were defined as genes expressed in at least one of the three somatic SAGE data sets (≥ 8 tags) and not expressed in the germline SAGE data set (≥ 1 tag). Larval/adult germline-specific genes (n = 675) were defined as the intersection of the germline-enriched genes [34], the germline expressed genes from SAGE analysis (≥ 1 tag) [35] and the class of 'strictly maternal' genes identified in the *C. elegans *embryonic transcriptome analysis by Baugh *et al. *[66].

### q-PCR analyses

The qPCR analyses were performed using a real-time PCR instrument (LightCycler 480 II; Roche Applied Sciences) with supplied reaction mix (LightCycler 480 SYBR Green 1 Master Mix; Roche Applied Sciences). Input DNA or DNA samples obtained by ChIP were amplified under conditions of 95°C for 10 min followed by 45 cycles of steps 95°C for 30 seconds, 60°C for 30 seconds and 72°C for 30 seconds. Percentage inputs were calculated by the formula:

% input=100×(2x(PCR efficiency)]^[−deltaCp(ChIP−Input))×dilution factor (ChIP)/dilution factor (input)×(% of extract used as input).

A file containing the sequences of the primers used is available (see Additional file 6).

### Note added in proof

Freter *et al. *[67] recently reported that RNA Pol II pSer2 is significantly reduced in a number of adult somatic and germline stem cell types, suggesting that the global repression of pSer2 that we observe in late stage *C. elegans *PGCs may be a conserved and common feature of pluripotent cells in many organisms.

## Competing interests

The authors declare that they have no competing interests.

## Authors' contributions

HF contributed to the design of the experiments, performed RNAi, immunofluorescence and ChIP experiments, contributed to the ChIP-seq gene set analyses, and wrote the manuscript. TT contributed to experimental design, performed immunofluorescence and qPCR experiments, and contributed to analyses of the results. AR performed bioinformatic and statistical analyses of the ChIP-seq data. TL performed RNAi and immunofluorescence experiments. PMC performed immunofluorescence experiments. HK provided antibodies used in immunofluorescence and ChIP experiments. SS contributed to the experimental design, data analyses and editing of the manuscript. WGK contributed to the overall experimental design, data analyses, and writing and editing of the manuscript. All authors read and approved the final manuscript.

## Supplementary Material

Additional file 1 **AMA-1 depletion by RNA interference (RNAi)**. Polymerase (Pol) II staining (using the 8WG16 antibody) and DAPI staining (middle and right, respectively) in *ama-1 (RNAi) *(top) and control embryos (bottom) are shown in grayscale with the merged image (left). Click here for file

Additional file 2 **Summary of H3K4me2, H3K36me3 and pSer2 dynamics in wild type and *mes-4 *Mutant embryonic germlines**. H3K4me2 (green bars) is maintained in the P-cells of both genotypes, and then largely erased after the primordial germ cells (PGCs) (Z2/Z3) are born. In WT, H3K4me2 levels remain reduced until hatching; in *mes-4 *embryos, there is strong reappearance observed long before hatching. The RNA polymerase (Pol) II C-terminal domain phosphoepitope detected by the H5 antibody (pSer2; red bars) is not observed in the P-cells of either genotype, but then appears strongly in the PGCs. Whereas in wild type this signal decreases to low levels in later stages, robust levels are maintained in the PGCs. The repression of pSer2 in the P cells is dependent on the maternal CCCH Zn finger proteins, OMA-1 and PIE-1 (black bar). H3K36me3 (blue bars) is maintained at high levels in all germline stages in WT embryos. The H3K36me3 that arrives in gamete chromatin (P0 to P1) is methyltransferase (MET)-1 dependent, as it is present in *mes-4 *embryos (bottom blue bar), but absent in *met-1;mes-4 *embryos (not shown). The maintenance of this methylation in the transcriptionally inactive P cells (pSer2; blue bars) is completely dependent on MES-4, as it is absent in *mes-4*. In later *mes-4 *PGCs, MET-1 dependent H3K36me3 appears in the PGCs, coincident with H3K4me2 (red bar) and the abnormal persistence of robust pSer2 (blue bar). Click here for file

Additional file 3 **Late stage embryo preparation for chromatin immunoprecipitation (ChIP)**. **(A) **Differential interference contras microscopy image showing a field of *met-1 *embryos after their isolation using the procedure described in Methods. **(B) **Immunofluorescence microscopy analysis of an estimated 'average' embryo stage of the *met-1 *embryos purified as in **(A)**, stained for H3K36me3. Red. DAPI; green; H3K36me3; blue, PGL-1 staining in merged image on left. H3K36me3 staining alone is shown in grayscale image on right. Arrow points to a primordial germ cell (PGC). Note that although there is enrichment for H3K36me3 in the PGC, significant levels are still detectable in the somatic nuclei. Click here for file

Additional file 4 **Quantitative (q)PCR analysis of H3K36me3 obtained by chromatin immunoprecipitation (ChIP) from mid-late staged *met-1 *embryos**. Chromatin obtained via ChIP with anti-H3K36me3 was analyzed by real time PCR using primers specific for genes expressed in germ cells (*htp-2, gld-1, pgl-1)*, genes expressed in somatic lineages (*myo-3, unc-52*), a gene expressed in both (*rpl-4*), an X-linked gene (*vha-15*) and a non-coding genomic region with no observed activity (non-coding). As was observed in the ChIP sequencing analyses, loci expressed in germ cells showed detectable maternal effect sterile (MES)-4-dependent H3K36me3, whereas those expressed only in soma show little signal above that observed for the non-coding region. Click here for file

Additional file 5 **Input chromatin H3K36me3 among gene sets analyzed**. The gene sets analyzed in Figure 10 **(A)**; germline-enriched genes, **(B) **ubiquitously expressed genes, **(C) **soma-specific genes and **(D) **larval/adult germline-specific genes, did not seem to be enriched for H3K36me3 in input chromatin from either *met-1 *or wild type late embryos. Click here for file

Additional file 6 **Primer sequences for quantitative (q)PCR**. The primer sequences used for pPCR validation, the genes to which they are targeted, the amplicon sizes and the amplicon coordinates or position within each target are indicated. Click here for file
