# Supplementary figures and images for: Trans-generational epigenetic regulation of C. elegans primordial germ cells
Source: Epigenetics Chromatin. 2010 Aug 12;3:15. doi: 10.1186/1756-8935-3-15 (PMC3146070; doi:10.1186/1756-8935-3-15)

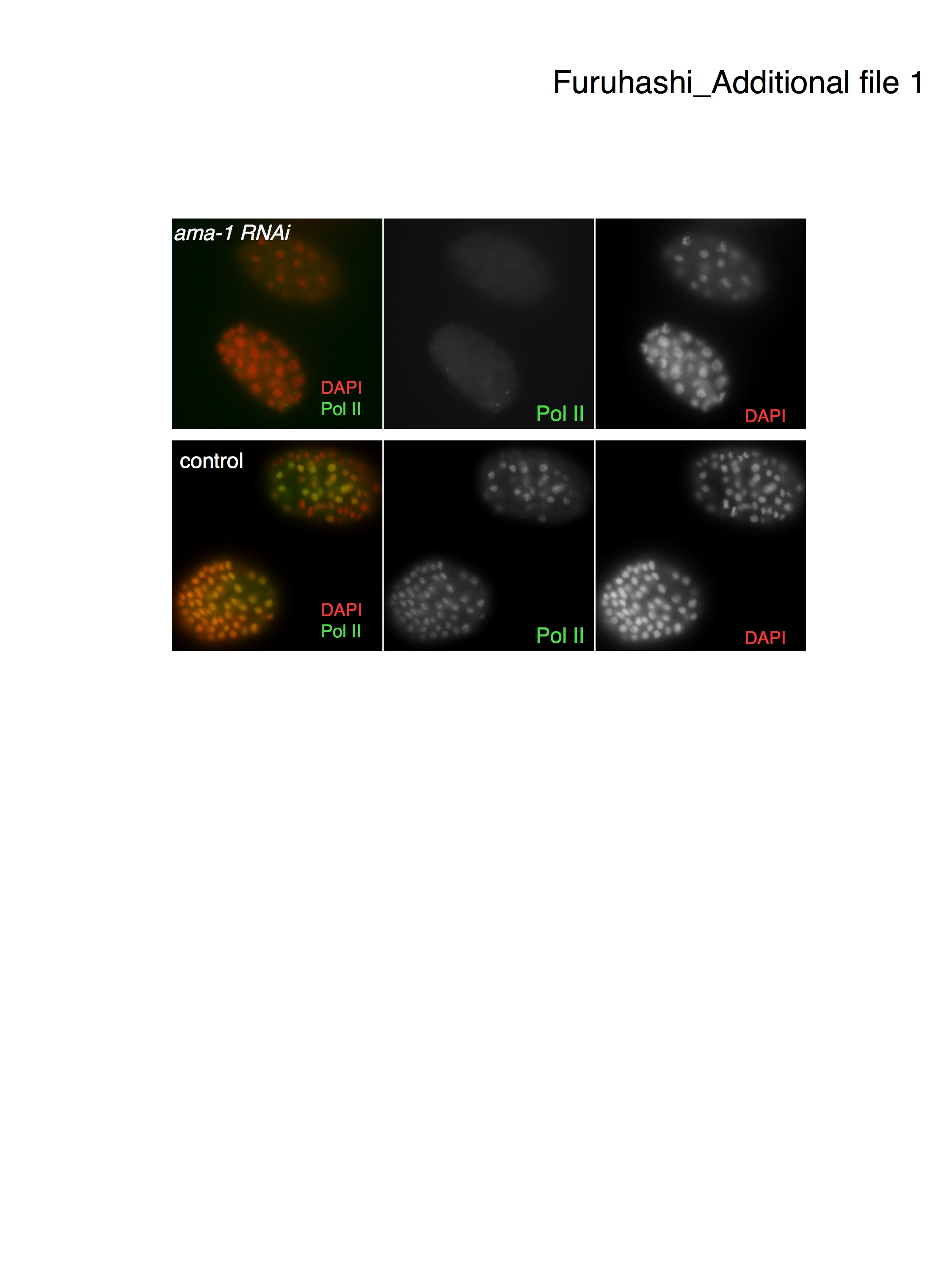

Supplement: Additional file 1 — AMA-1 depletion by RNA interference (RNAi). Polymerase (Pol) II staining (using the 8WG16 antibody) and DAPI staining (middle and right, respectively) in ama-1 (RNAi) (top) and control embryos (bottom) are shown in grayscale with the merged image (left). [file 1756-8935-3-15-S1.JPEG]

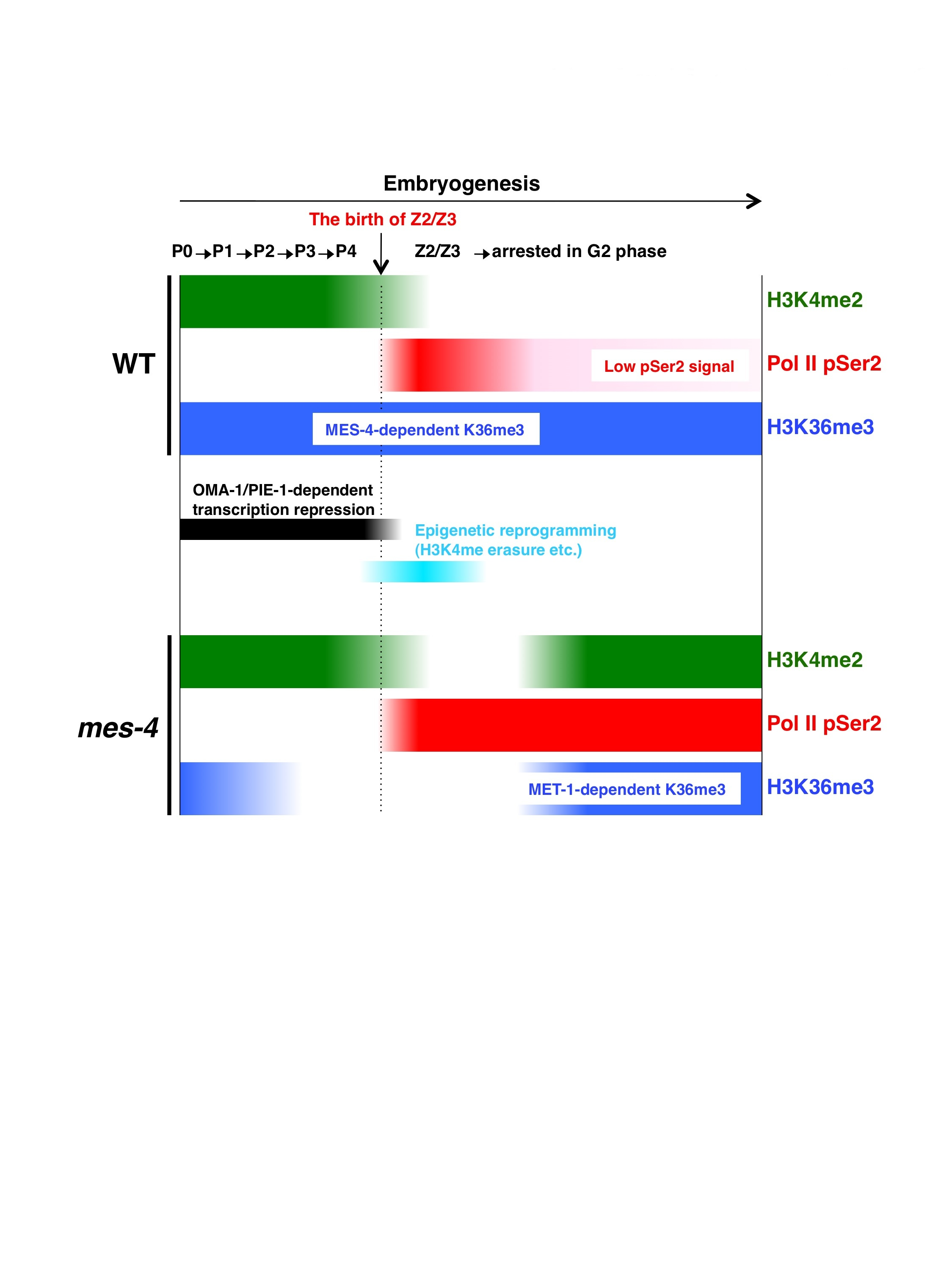

Supplement: Additional file 2 — Summary of H3K4me2, H3K36me3 and pSer2 dynamics in wild type and mes-4 Mutant embryonic germlines. H3K4me2 (green bars) is maintained in the P-cells of both genotypes, and then largely erased after the primordial germ cells (PGCs) (Z2/Z3) are born. In WT, H3K4me2 levels remain reduced until hatching; in mes-4 embryos, there is strong reappearance observed long before hatching. The RNA polymerase (Pol) II C-terminal domain phosphoepitope detected by the H5 antibody (pSer2; red bars) is not observed in the P-cells of either genotype, but then appears strongly in the PGCs. Whereas in wild type this signal decreases to low levels in later stages, robust levels are maintained in the PGCs. The repression of pSer2 in the P cells is dependent on the maternal CCCH Zn finger proteins, OMA-1 and PIE-1 (black bar). H3K36me3 (blue bars) is maintained at high levels in all germline stages in WT embryos. The H3K36me3 that arrives in gamete chromatin (P0 to P1) is methyltransferase (MET)-1 dependent, as it is present in mes-4 embryos (bottom blue bar), but absent in met-1;mes-4 embryos (not shown). The maintenance of this methylation in the transcriptionally inactive P cells (pSer2; blue bars) is completely dependent on MES-4, as it is absent in mes-4. In later mes-4 PGCs, MET-1 dependent H3K36me3 appears in the PGCs, coincident with H3K4me2 (red bar) and the abnormal persistence of robust pSer2 (blue bar). [file 1756-8935-3-15-S2.TIFF]

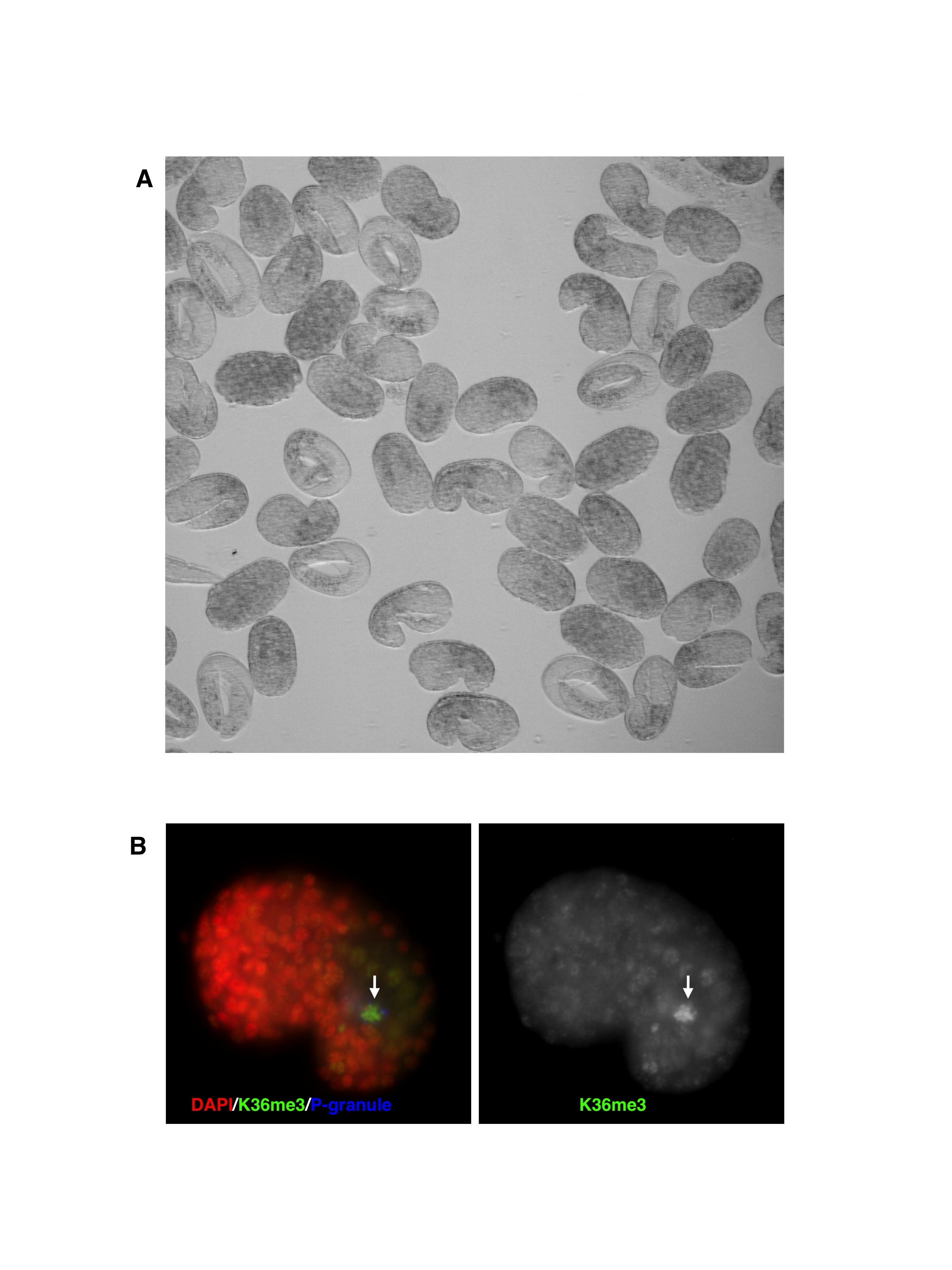

Supplement: Additional file 3 — Late stage embryo preparation for chromatin immunoprecipitation (ChIP). (A) Differential interference contras microscopy image showing a field of met-1 embryos after their isolation using the procedure described in Methods. (B) Immunofluorescence microscopy analysis of an estimated 'average' embryo stage of the met-1 embryos purified as in (A), stained for H3K36me3. Red. DAPI; green; H3K36me3; blue, PGL-1 staining in merged image on left. H3K36me3 staining alone is shown in grayscale image on right. Arrow points to a primordial germ cell (PGC). Note that although there is enrichment for H3K36me3 in the PGC, significant levels are still detectable in the somatic nuclei. [file 1756-8935-3-15-S3.TIFF]

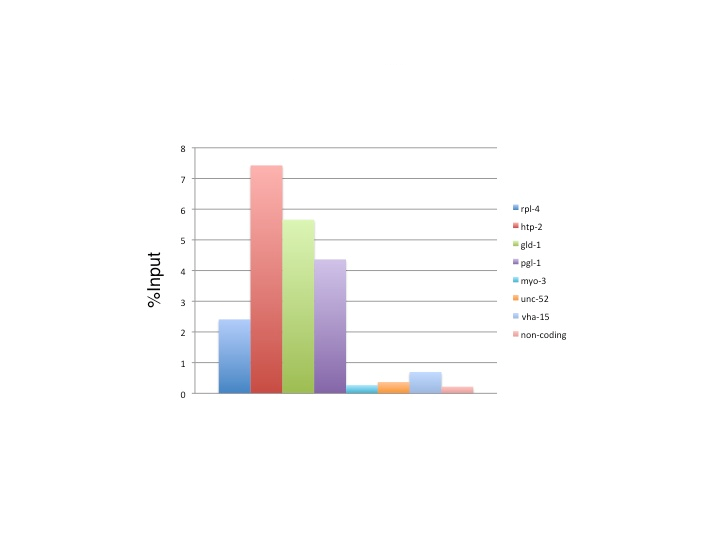

Supplement: Additional file 4 — Quantitative (q)PCR analysis of H3K36me3 obtained by chromatin immunoprecipitation (ChIP) from mid-late staged met-1 embryos. Chromatin obtained via ChIP with anti-H3K36me3 was analyzed by real time PCR using primers specific for genes expressed in germ cells (htp-2, gld-1, pgl-1), genes expressed in somatic lineages (myo-3, unc-52), a gene expressed in both (rpl-4), an X-linked gene (vha-15) and a non-coding genomic region with no observed activity (non-coding). As was observed in the ChIP sequencing analyses, loci expressed in germ cells showed detectable maternal effect sterile (MES)-4-dependent H3K36me3, whereas those expressed only in soma show little signal above that observed for the non-coding region. [file 1756-8935-3-15-S4.TIFF]

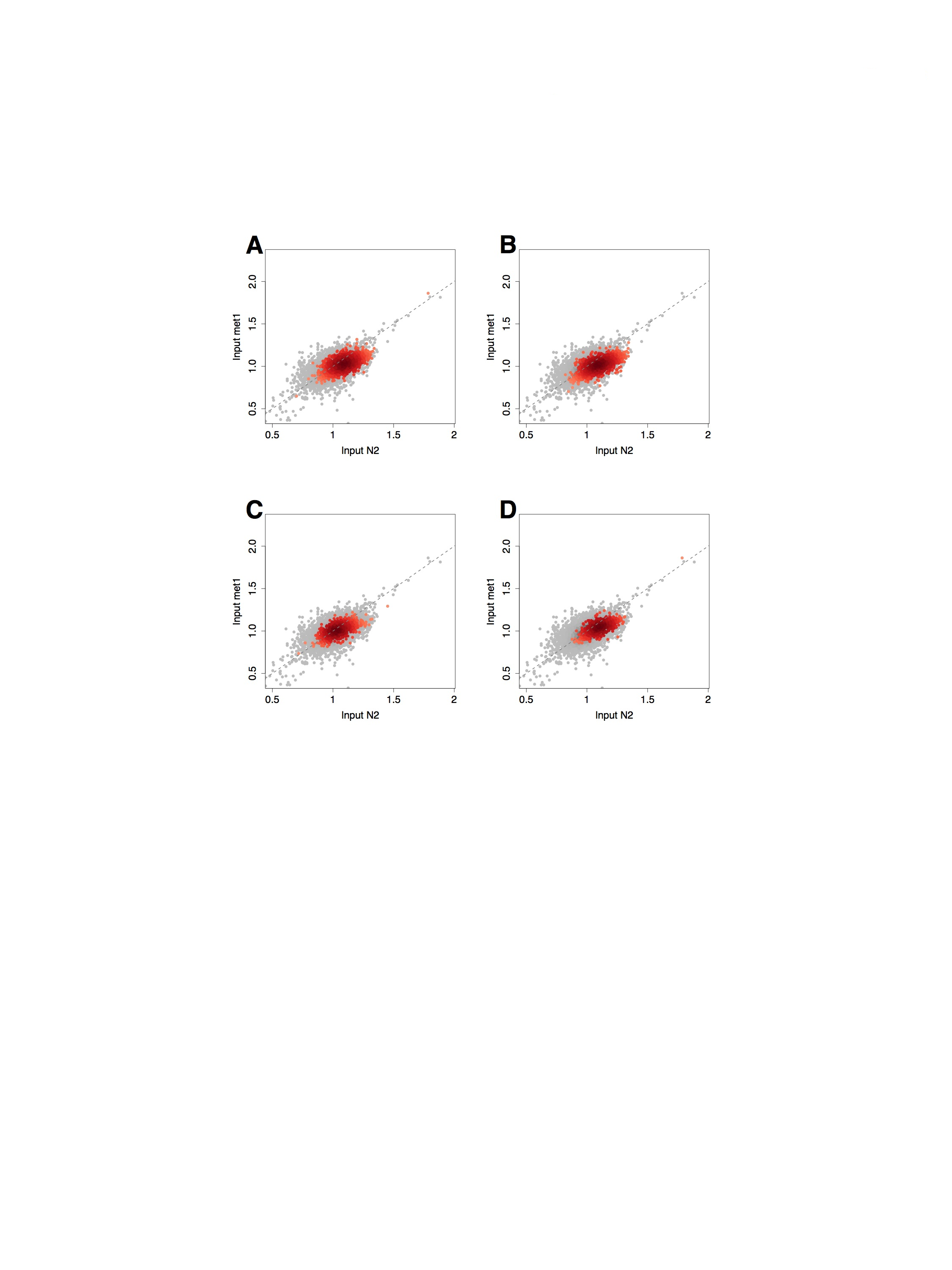

Supplement: Additional file 5 — Input chromatin H3K36me3 among gene sets analyzed. The gene sets analyzed in Figure 10 (A); germline-enriched genes, (B) ubiquitously expressed genes, (C) soma-specific genes and (D) larval/adult germline-specific genes, did not seem to be enriched for H3K36me3 in input chromatin from either met-1 or wild type late embryos. [file 1756-8935-3-15-S5.TIFF]
